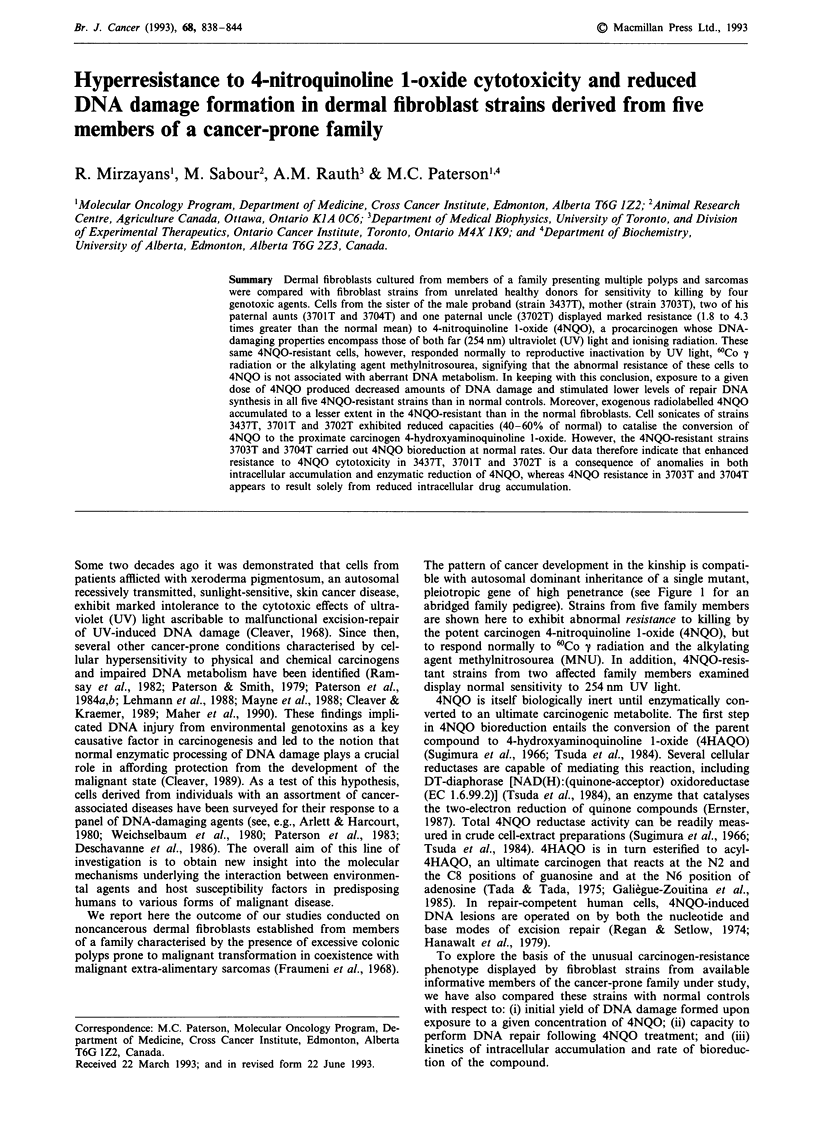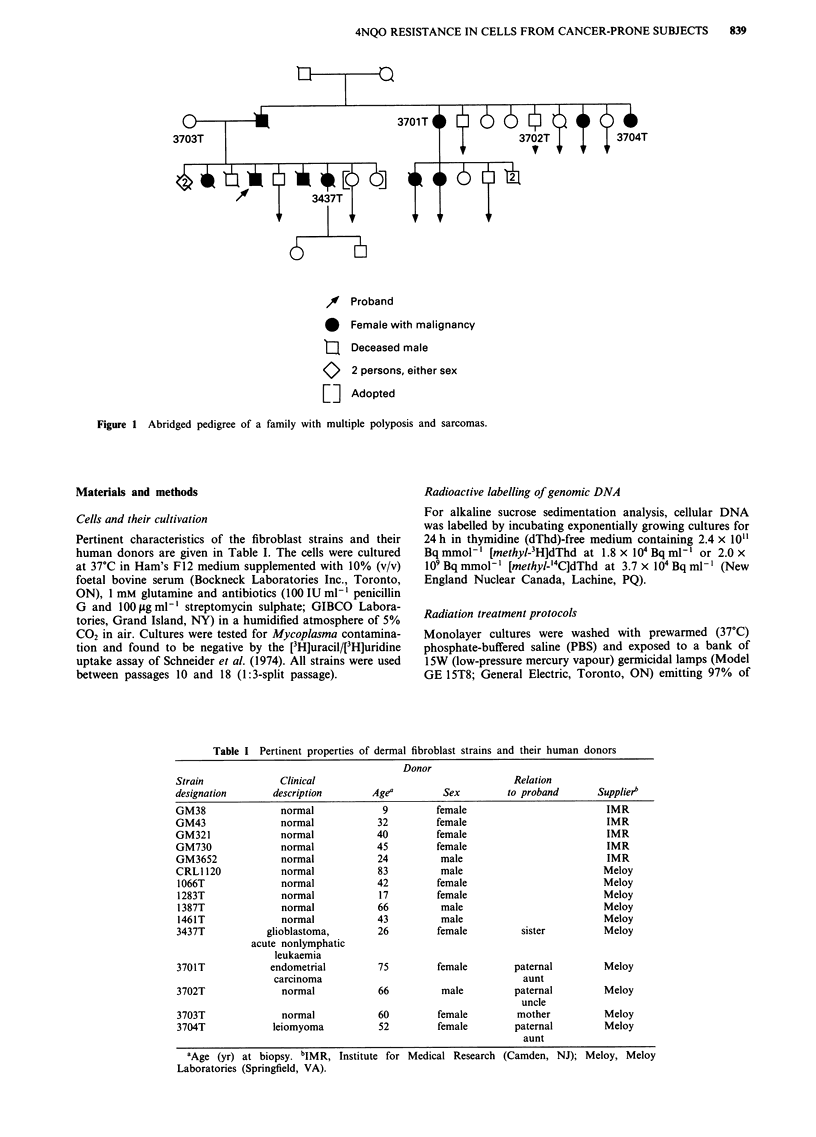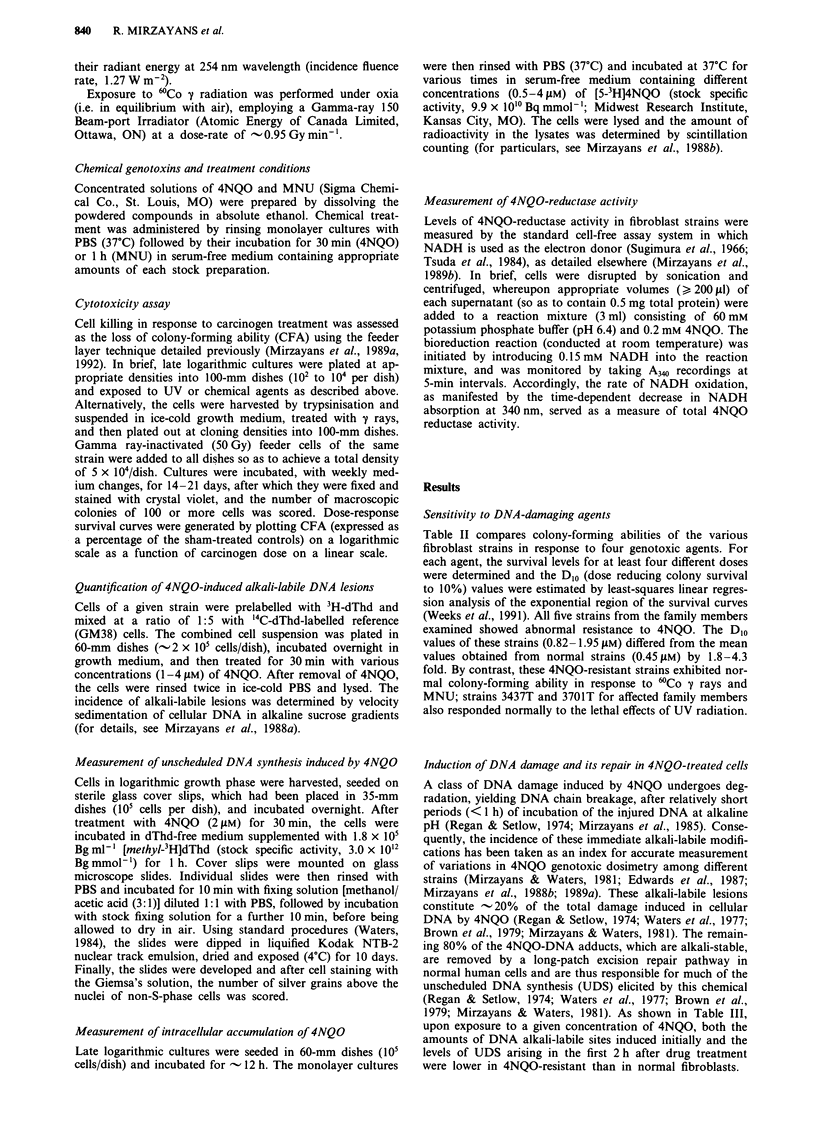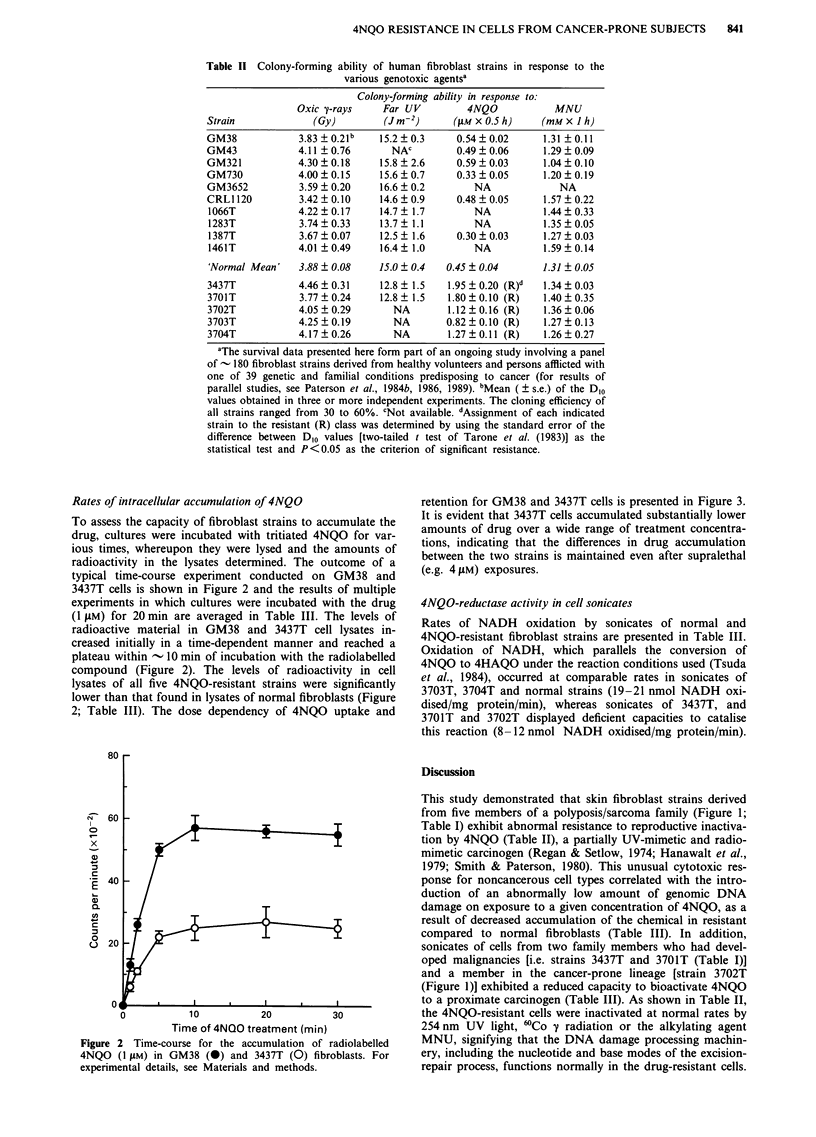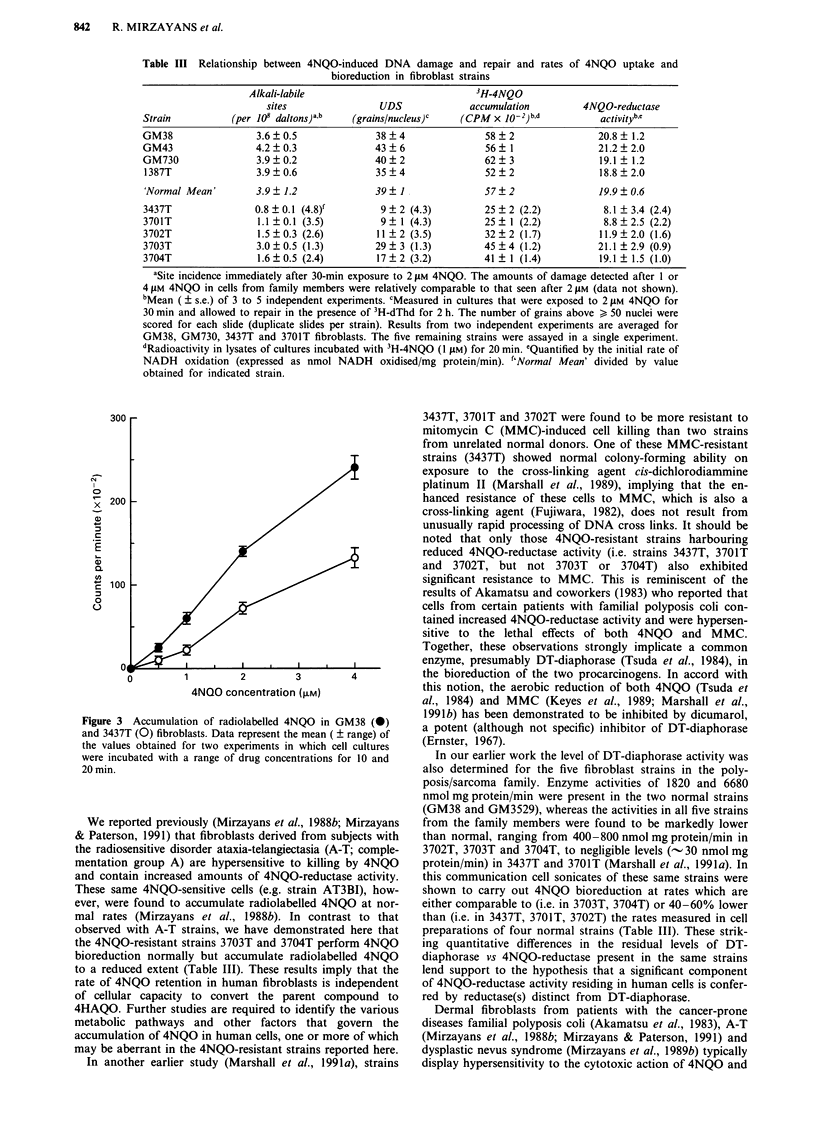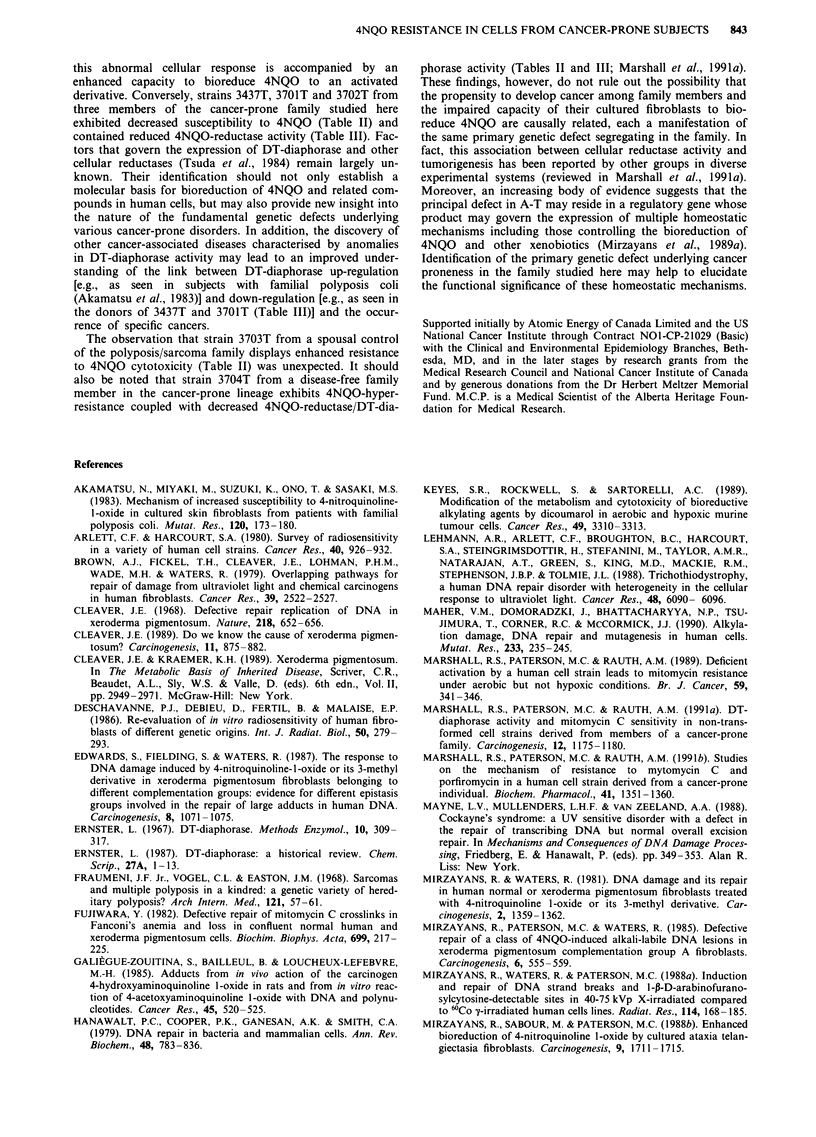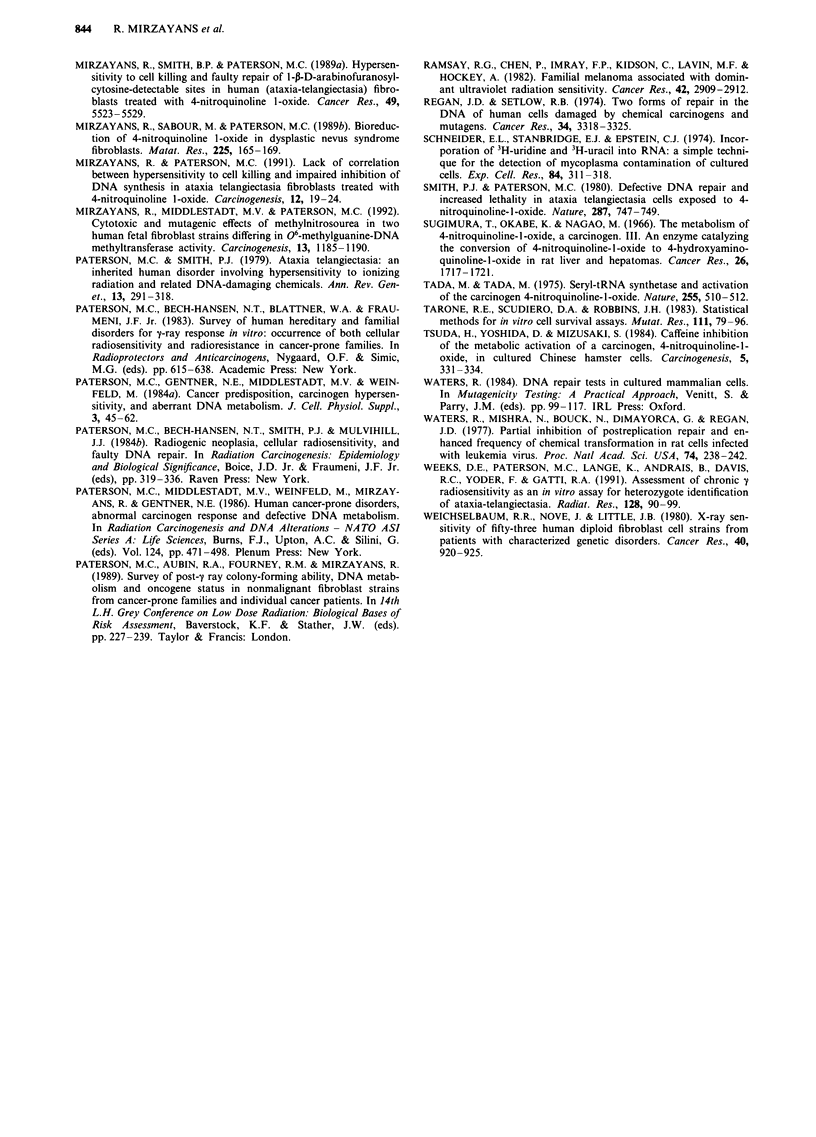# Hyperresistance to 4-nitroquinoline 1-oxide cytotoxicity and reduced DNA damage formation in dermal fibroblast strains derived from five members of a cancer-prone family.

**DOI:** 10.1038/bjc.1993.442

**Published:** 1993-11

**Authors:** R. Mirzayans, M. Sabour, A. M. Rauth, M. C. Paterson

**Affiliations:** Department of Medicine, Cross Cancer Institute, Edmonton, Alberta, Canada.

## Abstract

Dermal fibroblasts cultured from members of a family presenting multiple polyps and sarcomas were compared with fibroblast strains from unrelated healthy donors for sensitivity to killing by four genotoxic agents. Cells from the sister of the male proband (strain 3437T), mother (strain 3703T), two of his paternal aunts (3701T and 3704T) and one paternal uncle (3702T) displayed marked resistance (1.8 to 4.3 times greater than the normal mean) to 4-nitroquinoline 1-oxide (4NQO), a procarcinogen whose DNA-damaging properties encompass those of both far (254 nm) ultraviolet (UV) light and ionising radiation. These same 4NQO-resistant cells, however, responded normally to reproductive inactivation by UV light, 60Co gamma radiation or the alkylating agent methylnitrosourea, signifying that the abnormal resistance of these cells to 4NQO is not associated with aberrant DNA metabolism. In keeping with this conclusion, exposure to a given dose of 4NQO produced decreased amounts of DNA damage and stimulated lower levels of repair DNA synthesis in all five 4NQO-resistant strains than in normal controls. Moreover, exogenous radiolabelled 4NQO accumulated to a lesser extent in the 4NQO-resistant than in the normal fibroblasts. Cell sonicates of strains 3437T, 3701T and 3702T exhibited reduced capacities (40-60% of normal) to catalise the conversion of 4NQO to the proximate carcinogen 4-hydroxyaminoquinoline 1-oxide. However, the 4NQO-resistant strains 3703T and 3704T carried out 4NQO bioreduction at normal rates. Our data therefore indicate that enhanced resistance to 4NQO cytotoxicity in 3437T, 3701T and 3702T is a consequence of anomalies in both intracellular accumulation and enzymatic reduction of 4NQO, whereas 4NQO resistance in 3703T and 3704T appears to result solely from reduced intracellular drug accumulation.


Br. J. Cancer (1993), 68, 838–844 © Macmillan Press Ltd., 1993

### Hyperresistance to 4-nitroquinoline 1-oxide cytotoxicity and reduced DNA damage formation in dermal fibroblast strains derived from five members of a cancer-prone family

R. Mirzayans[1], M. Sabour[2], A.M. Rauth[3] & M.C. Paterson[1,4]

[1]*Molecular Oncology Program, Department of Medicine, Cross Cancer Institute, Edmonton, Alberta T6G 1Z2;* [2]*Animal Research Centre, Agriculture Canada, Ottawa, Ontario K1A 0C6;* [3]*Department of Medical Biophysics, University of Toronto, and Division of Experimental Therapeutics, Ontario Cancer Institute, Toronto, Ontario M4X 1K9; and* [4]*Department of Biochemistry, University of Alberta, Edmonton, Alberta T6G 2Z3, Canada.*

**Summary** Dermal fibroblasts cultured from members of a family presenting multiple polyps and sarcomas were compared with fibroblast strains from unrelated healthy donors for sensitivity to killing by four genotoxic agents. Cells from the sister of the male proband (strain 3437T), mother (strain 3703T), two of his paternal aunts (3701T and 3704T) and one paternal uncle (3702T) displayed marked resistance (1.8 to 4.3 times greater than the normal mean) to 4-nitroquinoline 1-oxide (4NQO), a procarcinogen whose DNA-damaging properties encompass those of both far (254 nm) ultraviolet (UV) light and ionising radiation. These same 4NQO-resistant cells, however, responded normally to reproductive inactivation by UV light, $^{60}$Co γ radiation or the alkylating agent methylnitrosourea, signifying that the abnormal resistance of these cells to 4NQO is not associated with aberrant DNA metabolism. In keeping with this conclusion, exposure to a given dose of 4NQO produced decreased amounts of DNA damage and stimulated lower levels of repair DNA synthesis in all five 4NQO-resistant strains than in normal controls. Moreover, exogenous radiolabelled 4NQO accumulated to a lesser extent in the 4NQO-resistant than in the normal fibroblasts. Cell sonicates of strains 3437T, 3701T and 3702T exhibited reduced capacities (40–60% of normal) to catalise the conversion of 4NQO to the proximate carcinogen 4-hydroxyaminoquinoline 1-oxide. However, the 4NQO-resistant strains 3703T and 3704T carried out 4NQO bioreduction at normal rates. Our data therefore indicate that enhanced resistance to 4NQO cytotoxicity in 3437T, 3701T and 3702T is a consequence of anomalies in both intracellular accumulation and enzymatic reduction of 4NQO, whereas 4NQO resistance in 3703T and 3704T appears to result solely from reduced intracellular drug accumulation.

Some two decades ago it was demonstrated that cells from patients afflicted with xeroderma pigmentosum, an autosomal recessively transmitted, sunlight-sensitive, skin cancer disease, exhibit marked intolerance to the cytotoxic effects of ultraviolet (UV) light ascribable to malfunctional excision-repair of UV-induced DNA damage (Cleaver, 1968). Since then, several other cancer-prone conditions characterised by cellular hypersensitivity to physical and chemical carcinogens and impaired DNA metabolism have been identified (Ramsay *et al.*, 1982; Paterson & Smith, 1979; Paterson *et al.*, 1984*a,b*; Lehmann *et al.*, 1988; Mayne *et al.*, 1988; Cleaver & Kraemer, 1989; Maher *et al.*, 1990). These findings implicated DNA injury from environmental genotoxins as a key causative factor in carcinogenesis and led to the notion that normal enzymatic processing of DNA damage plays a crucial role in affording protection from the development of the malignant state (Cleaver, 1989). As a test of this hypothesis, cells derived from individuals with an assortment of cancer-associated diseases have been surveyed for their response to a panel of DNA-damaging agents (see, e.g., Arlett & Harcourt, 1980; Weichselbaum *et al.*, 1980; Paterson *et al.*, 1983; Deschavanne *et al.*, 1986). The overall aim of this line of investigation is to obtain new insight into the molecular mechanisms underlying the interaction between environmental agents and host susceptibility factors in predisposing humans to various forms of malignant disease.

We report here the outcome of our studies conducted on noncancerous dermal fibroblasts established from members of a family characterised by the presence of excessive colonic polyps prone to malignant transformation in coexistence with malignant extra-alimentary sarcomas (Fraumeni *et al.*, 1968). The pattern of cancer development in the kinship is compatible with autosomal dominant inheritance of a single mutant, pleiotropic gene of high penetrance (see Figure 1 for an abridged family pedigree). Strains from five family members are shown here to exhibit abnormal *resistance* to killing by the potent carcinogen 4-nitroquinoline 1-oxide (4NQO), but to respond normally to $^{60}$Co γ radiation and the alkylating agent methylnitrosourea (MNU). In addition, 4NQO-resistant strains from two affected family members examined display normal sensitivity to 254 nm UV light.

4NQO is itself biologically inert until enzymatically converted to an ultimate carcinogenic metabolite. The first step in 4NQO bioreduction entails the conversion of the parent compound to 4-hydroxyaminoquinoline 1-oxide (4HAQO) (Sugimura *et al.*, 1966; Tsuda *et al.*, 1984). Several cellular reductases are capable of mediating this reaction, including DT-diaphorase [NAD(H):(quinone-acceptor) oxidoreductase (EC 1.6.99.2)] (Tsuda *et al.*, 1984), an enzyme that catalyses the two-electron reduction of quinone compounds (Ernster, 1987). Total 4NQO reductase activity can be readily measured in crude cell-extract preparations (Sugimura *et al.*, 1966; Tsuda *et al.*, 1984). 4HAQO is in turn esterified to acyl-4HAQO, an ultimate carcinogen that reacts at the N2 and the C8 positions of guanosine and at the N6 position of adenosine (Tada & Tada, 1975; Galiègue-Zouitina *et al.*, 1985). In repair-competent human cells, 4NQO-induced DNA lesions are operated on by both the nucleotide and base modes of excision repair (Regan & Setlow, 1974; Hanawalt *et al.*, 1979).

To explore the basis of the unusual carcinogen-resistance phenotype displayed by fibroblast strains from available informative members of the cancer-prone family under study, we have also compared these strains with normal controls with respect to: (i) initial yield of DNA damage formed upon exposure to a given concentration of 4NQO; (ii) capacity to perform DNA repair following 4NQO treatment; and (iii) kinetics of intracellular accumulation and rate of bioreduction of the compound.

Correspondence: M.C. Paterson, Molecular Oncology Program, Department of Medicine, Cross Cancer Institute, Edmonton, Alberta T6G 1Z2, Canada.
Received 22 March 1993; and in revised form 22 June 1993.

**Figure 1** Abridged pedigree of a family with multiple polyposis and sarcomas.

### Materials and methods

### *Cells and their cultivation*

Pertinent characteristics of the fibroblast strains and their human donors are given in Table I. The cells were cultured at 37°C in Ham's F12 medium supplemented with 10% (v/v) foetal bovine serum (Bockneck Laboratories Inc., Toronto, ON), 1 mM glutamine and antibiotics (100 IU $ml^{-1}$ penicillin G and 100 $\mu g\ ml^{-1}$ streptomycin sulphate; GIBCO Laboratories, Grand Island, NY) in a humidified atmosphere of 5% $CO_2$ in air. Cultures were tested for *Mycoplasma* contamination and found to be negative by the [$^3$H]uracil/[$^3$H]uridine uptake assay of Schneider *et al.* (1974). All strains were used between passages 10 and 18 (1:3-split passage).

### *Radioactive labelling of genomic DNA*

For alkaline sucrose sedimentation analysis, cellular DNA was labelled by incubating exponentially growing cultures for 24 h in thymidine (dThd)-free medium containing $2.4 \times 10^{11}$ Bq $mmol^{-1}$ [*methyl*-$^3$H]dThd at $1.8 \times 10^4$ Bq $ml^{-1}$ or $2.0 \times 10^9$ Bq $mmol^{-1}$ [*methyl*-$^{14}$C]dThd at $3.7 \times 10^4$ Bq $ml^{-1}$ (New England Nuclear Canada, Lachine, PQ).

### *Radiation treatment protocols*

Monolayer cultures were washed with prewarmed (37°C) phosphate-buffered saline (PBS) and exposed to a bank of 15W (low-pressure mercury vapour) germicidal lamps (Model GE 15T8; General Electric, Toronto, ON) emitting 97% of

**Table I** Pertinent properties of dermal fibroblast strains and their human donors

| Strain designation | Clinical description | Donor Age[a] | Sex | Relation to proband | Supplier[b] |
|---|---|---|---|---|---|
| GM38 | normal | 9 | female | | IMR |
| GM43 | normal | 32 | female | | IMR |
| GM321 | normal | 40 | female | | IMR |
| GM730 | normal | 45 | female | | IMR |
| GM3652 | normal | 24 | male | | IMR |
| CRL1120 | normal | 83 | male | | Meloy |
| 1066T | normal | 42 | female | | Meloy |
| 1283T | normal | 17 | female | | Meloy |
| 1387T | normal | 66 | male | | Meloy |
| 1461T | normal | 43 | male | | Meloy |
| 3437T | glioblastoma, acute nonlymphatic leukaemia | 26 | female | sister | Meloy |
| 3701T | endometrial carcinoma | 75 | female | paternal aunt | Meloy |
| 3702T | normal | 66 | male | paternal uncle | Meloy |
| 3703T | normal | 60 | female | mother | Meloy |
| 3704T | leiomyoma | 52 | female | paternal aunt | Meloy |

[a]Age (yr) at biopsy. [b]IMR, Institute for Medical Research (Camden, NJ); Meloy, Meloy Laboratories (Springfield, VA).

their radiant energy at 254 nm wavelength (incidence fluence rate, 1.27 W m$^{-2}$).

Exposure to $^{60}$Co γ radiation was performed under oxia (i.e. in equilibrium with air), employing a Gamma-ray 150 Beam-port Irradiator (Atomic Energy of Canada Limited, Ottawa, ON) at a dose-rate of ~0.95 Gy min$^{-1}$.

*Chemical genotoxins and treatment conditions*

Concentrated solutions of 4NQO and MNU (Sigma Chemical Co., St. Louis, MO) were prepared by dissolving the powdered compounds in absolute ethanol. Chemical treatment was administered by rinsing monolayer cultures with PBS (37°C) followed by their incubation for 30 min (4NQO) or 1 h (MNU) in serum-free medium containing appropriate amounts of each stock preparation.

*Cytotoxicity assay*

Cell killing in response to carcinogen treatment was assessed as the loss of colony-forming ability (CFA) using the feeder layer technique detailed previously (Mirzayans *et al.*, 1989*a*, 1992). In brief, late logarithmic cultures were plated at appropriate densities into 100-mm dishes ($10^2$ to $10^4$ per dish) and exposed to UV or chemical agents as described above. Alternatively, the cells were harvested by trypsinisation and suspended in ice-cold growth medium, treated with γ rays, and then plated out at cloning densities into 100-mm dishes. Gamma ray-inactivated (50 Gy) feeder cells of the same strain were added to all dishes so as to achieve a total density of 5 × $10^4$/dish. Cultures were incubated, with weekly medium changes, for 14–21 days, after which they were fixed and stained with crystal violet, and the number of macroscopic colonies of 100 or more cells was scored. Dose-response survival curves were generated by plotting CFA (expressed as a percentage of the sham-treated controls) on a logarithmic scale as a function of carcinogen dose on a linear scale.

*Quantification of 4NQO-induced alkali-labile DNA lesions*

Cells of a given strain were prelabelled with $^3$H-dThd and mixed at a ratio of 1:5 with $^{14}$C-dThd-labelled reference (GM38) cells. The combined cell suspension was plated in 60-mm dishes (~2 × $10^5$ cells/dish), incubated overnight in growth medium, and then treated for 30 min with various concentrations (1–4 μM) of 4NQO. After removal of 4NQO, the cells were rinsed twice in ice-cold PBS and lysed. The incidence of alkali-labile lesions was determined by velocity sedimentation of cellular DNA in alkaline sucrose gradients (for details, see Mirzayans *et al.*, 1988*a*).

*Measurement of unscheduled DNA synthesis induced by 4NQO*

Cells in logarithmic growth phase were harvested, seeded on sterile glass cover slips, which had been placed in 35-mm dishes ($10^5$ cells per dish), and incubated overnight. After treatment with 4NQO (2 μM) for 30 min, the cells were incubated in dThd-free medium supplemented with 1.8 × $10^5$ Bg ml$^{-1}$ [*methyl*-$^3$H]dThd (stock specific activity, 3.0 × $10^{12}$ Bg mmol$^{-1}$) for 1 h. Cover slips were mounted on glass microscope slides. Individual slides were then rinsed with PBS and incubated for 10 min with fixing solution [methanol/acetic acid (3:1)] diluted 1:1 with PBS, followed by incubation with stock fixing solution for a further 10 min, before being allowed to dry in air. Using standard procedures (Waters, 1984), the slides were dipped in liquified Kodak NTB-2 nuclear track emulsion, dried and exposed (4°C) for 10 days. Finally, the slides were developed and after cell staining with the Giemsa's solution, the number of silver grains above the nuclei of non-S-phase cells was scored.

*Measurement of intracellular accumulation of 4NQO*

Late logarithmic cultures were seeded in 60-mm dishes ($10^5$ cells/dish) and incubated for ~12 h. The monolayer cultures were then rinsed with PBS (37°C) and incubated at 37°C for various times in serum-free medium containing different concentrations (0.5–4 μM) of [5-$^3$H]4NQO (stock specific activity, 9.9 × $10^{10}$ Bq mmol$^{-1}$; Midwest Research Institute, Kansas City, MO). The cells were lysed and the amount of radioactivity in the lysates was determined by scintillation counting (for particulars, see Mirzayans *et al.*, 1988*b*).

*Measurement of 4NQO-reductase activity*

Levels of 4NQO-reductase activity in fibroblast strains were measured by the standard cell-free assay system in which NADH is used as the electron donor (Sugimura *et al.*, 1966; Tsuda *et al.*, 1984), as detailed elsewhere (Mirzayans *et al.*, 1989*b*). In brief, cells were disrupted by sonication and centrifuged, whereupon appropriate volumes (⩾ 200 μl) of each supernatant (so as to contain 0.5 mg total protein) were added to a reaction mixture (3 ml) consisting of 60 mM potassium phosphate buffer (pH 6.4) and 0.2 mM 4NQO. The bioreduction reaction (conducted at room temperature) was initiated by introducing 0.15 mM NADH into the reaction mixture, and was monitored by taking $A_{340}$ recordings at 5-min intervals. Accordingly, the rate of NADH oxidation, as manifested by the time-dependent decrease in NADH absorption at 340 nm, served as a measure of total 4NQO reductase activity.

### Results

*Sensitivity to DNA-damaging agents*

Table II compares colony-forming abilities of the various fibroblast strains in response to four genotoxic agents. For each agent, the survival levels for at least four different doses were determined and the $D_{10}$ (dose reducing colony survival to 10%) values were estimated by least-squares linear regression analysis of the exponential region of the survival curves (Weeks *et al.*, 1991). All five strains from the family members examined showed abnormal resistance to 4NQO. The $D_{10}$ values of these strains (0.82–1.95 μM) differed from the mean values obtained from normal strains (0.45 μM) by 1.8–4.3 fold. By contrast, these 4NQO-resistant strains exhibited normal colony-forming ability in response to $^{60}$Co γ rays and MNU; strains 3437T and 3701T for affected family members also responded normally to the lethal effects of UV radiation.

*Induction of DNA damage and its repair in 4NQO-treated cells*

A class of DNA damage induced by 4NQO undergoes degradation, yielding DNA chain breakage, after relatively short periods (< 1 h) of incubation of the injured DNA at alkaline pH (Regan & Setlow, 1974; Mirzayans *et al.*, 1985). Consequently, the incidence of these immediate alkali-labile modifications has been taken as an index for accurate measurement of variations in 4NQO genotoxic dosimetry among different strains (Mirzayans & Waters, 1981; Edwards *et al.*, 1987; Mirzayans *et al.*, 1988*b*; 1989*a*). These alkali-labile lesions constitute ~20% of the total damage induced in cellular DNA by 4NQO (Regan & Setlow, 1974; Waters *et al.*, 1977; Brown *et al.*, 1979; Mirzayans & Waters, 1981). The remaining 80% of the 4NQO-DNA adducts, which are alkali-stable, are removed by a long-patch excision repair pathway in normal human cells and are thus responsible for much of the unscheduled DNA synthesis (UDS) elicited by this chemical (Regan & Setlow, 1974; Waters *et al.*, 1977; Brown *et al.*, 1979; Mirzayans & Waters, 1981). As shown in Table III, upon exposure to a given concentration of 4NQO, both the amounts of DNA alkali-labile sites induced initially and the levels of UDS arising in the first 2 h after drug treatment were lower in 4NQO-resistant than in normal fibroblasts.

**Table II** Colony-forming ability of human fibroblast strains in response to the various genotoxic agents[a]

| Strain | *Oxic γ-rays (Gy)* | *Far UV ($J m^{-2}$)* | *4NQO (μM × 0.5 h)* | *MNU (mM × 1 h)* |
|---|---|---|---|---|
| | *Colony-forming ability in response to:* | | | |
| GM38 | 3.83 ± 0.21[b] | 15.2 ± 0.3 | 0.54 ± 0.02 | 1.31 ± 0.11 |
| GM43 | 4.11 ± 0.76 | NA[c] | 0.49 ± 0.06 | 1.29 ± 0.09 |
| GM321 | 4.30 ± 0.18 | 15.8 ± 2.6 | 0.59 ± 0.03 | 1.04 ± 0.10 |
| GM730 | 4.00 ± 0.15 | 15.6 ± 0.7 | 0.33 ± 0.05 | 1.20 ± 0.19 |
| GM3652 | 3.59 ± 0.20 | 16.6 ± 0.2 | NA | NA |
| CRL1120 | 3.42 ± 0.10 | 14.6 ± 0.9 | 0.48 ± 0.05 | 1.57 ± 0.22 |
| 1066T | 4.22 ± 0.17 | 14.7 ± 1.7 | NA | 1.44 ± 0.33 |
| 1283T | 3.74 ± 0.33 | 13.7 ± 1.1 | NA | 1.35 ± 0.05 |
| 1387T | 3.67 ± 0.07 | 12.5 ± 1.6 | 0.30 ± 0.03 | 1.27 ± 0.03 |
| 1461T | 4.01 ± 0.49 | 16.4 ± 1.0 | NA | 1.59 ± 0.14 |
| *'Normal Mean'* | *3.88 ± 0.08* | *15.0 ± 0.4* | *0.45 ± 0.04* | *1.31 ± 0.05* |
| 3437T | 4.46 ± 0.31 | 12.8 ± 1.5 | 1.95 ± 0.20 (R)[d] | 1.34 ± 0.03 |
| 3701T | 3.77 ± 0.24 | 12.8 ± 1.5 | 1.80 ± 0.10 (R) | 1.40 ± 0.35 |
| 3702T | 4.05 ± 0.29 | NA | 1.12 ± 0.16 (R) | 1.36 ± 0.06 |
| 3703T | 4.25 ± 0.19 | NA | 0.82 ± 0.10 (R) | 1.27 ± 0.13 |
| 3704T | 4.17 ± 0.26 | NA | 1.27 ± 0.11 (R) | 1.26 ± 0.27 |

[a]The survival data presented here form part of an ongoing study involving a panel of ~180 fibroblast strains derived from healthy volunteers and persons afflicted with one of 39 genetic and familial conditions predisposing to cancer (for results of parallel studies, see Paterson *et al.*, 1984*b*, 1986, 1989). [b]Mean (± s.e.) of the $D_{10}$ values obtained in three or more independent experiments. The cloning efficiency of all strains ranged from 30 to 60%. [c]Not available. [d]Assignment of each indicated strain to the resistant (R) class was determined by using the standard error of the difference between $D_{10}$ values [two-tailed *t* test of Tarone *et al.* (1983)] as the statistical test and $P < 0.05$ as the criterion of significant resistance.

### *Rates of intracellular accumulation of 4NQO*

To assess the capacity of fibroblast strains to accumulate the drug, cultures were incubated with tritiated 4NQO for various times, whereupon they were lysed and the amounts of radioactivity in the lysates determined. The outcome of a typical time-course experiment conducted on GM38 and 3437T cells is shown in Figure 2 and the results of multiple experiments in which cultures were incubated with the drug (1 μM) for 20 min are averaged in Table III. The levels of radioactive material in GM38 and 3437T cell lysates increased initially in a time-dependent manner and reached a plateau within ~10 min of incubation with the radiolabelled compound (Figure 2). The levels of radioactivity in cell lysates of all five 4NQO-resistant strains were significantly lower than that found in lysates of normal fibroblasts (Figure 2; Table III). The dose dependency of 4NQO uptake and retention for GM38 and 3437T cells is presented in Figure 3. It is evident that 3437T cells accumulated substantially lower amounts of drug over a wide range of treatment concentrations, indicating that the differences in drug accumulation between the two strains is maintained even after supralethal (e.g. 4 μM) exposures.

**Figure 2** Time-course for the accumulation of radiolabelled 4NQO (1 μM) in GM38 (●) and 3437T (○) fibroblasts. For experimental details, see Materials and methods.

### *4NQO-reductase activity in cell sonicates*

Rates of NADH oxidation by sonicates of normal and 4NQO-resistant fibroblast strains are presented in Table III. Oxidation of NADH, which parallels the conversion of 4NQO to 4HAQO under the reaction conditions used (Tsuda *et al.*, 1984), occurred at comparable rates in sonicates of 3703T, 3704T and normal strains (19–21 nmol NADH oxidised/mg protein/min), whereas sonicates of 3437T, and 3701T and 3702T displayed deficient capacities to catalise this reaction (8–12 nmol NADH oxidised/mg protein/min).

### Discussion

This study demonstrated that skin fibroblast strains derived from five members of a polyposis/sarcoma family (Figure 1; Table I) exhibit abnormal resistance to reproductive inactivation by 4NQO (Table II), a partially UV-mimetic and radiomimetic carcinogen (Regan & Setlow, 1974; Hanawalt *et al.*, 1979; Smith & Paterson, 1980). This unusual cytotoxic response for noncancerous cell types correlated with the introduction of an abnormally low amount of genomic DNA damage on exposure to a given concentration of 4NQO, as a result of decreased accumulation of the chemical in resistant compared to normal fibroblasts (Table III). In addition, sonicates of cells from two family members who had developed malignancies [i.e. strains 3437T and 3701T (Table I)] and a member in the cancer-prone lineage [strain 3702T (Figure 1)] exhibited a reduced capacity to bioactivate 4NQO to a proximate carcinogen (Table III). As shown in Table II, the 4NQO-resistant cells were inactivated at normal rates by 254 nm UV light, $^{60}Co$ γ radiation or the alkylating agent MNU, signifying that the DNA damage processing machinery, including the nucleotide and base modes of the excision-repair process, functions normally in the drug-resistant cells.

**Table III** Relationship between 4NQO-induced DNA damage and repair and rates of 4NQO uptake and bioreduction in fibroblast strains

| *Strain* | *Alkali-labile sites (per $10^8$ daltons)*[a,b] | *UDS (grains/nucleus)*[c] | *$^3$H-4NQO accumulation (CPM × $10^{-2}$)*[b,d] | *4NQO-reductase activity*[b,e] |
|---|---|---|---|---|
| GM38 | 3.6 ± 0.5 | 38 ± 4 | 58 ± 2 | 20.8 ± 1.2 |
| GM43 | 4.2 ± 0.3 | 43 ± 6 | 56 ± 1 | 21.2 ± 2.0 |
| GM730 | 3.9 ± 0.2 | 40 ± 2 | 62 ± 3 | 19.1 ± 1.2 |
| 1387T | 3.9 ± 0.6 | 35 ± 4 | 52 ± 2 | 18.8 ± 2.0 |
| *'Normal Mean'* | *3.9 ± 1.2* | *39 ± 1* | *57 ± 2* | *19.9 ± 0.6* |
| 3437T | 0.8 ± 0.1 (4.8)[f] | 9 ± 2 (4.3) | 25 ± 2 (2.2) | 8.1 ± 3.4 (2.4) |
| 3701T | 1.1 ± 0.1 (3.5) | 9 ± 1 (4.3) | 25 ± 1 (2.2) | 8.8 ± 2.5 (2.2) |
| 3702T | 1.5 ± 0.3 (2.6) | 11 ± 2 (3.5) | 32 ± 2 (1.7) | 11.9 ± 2.0 (1.6) |
| 3703T | 3.0 ± 0.5 (1.3) | 29 ± 3 (1.3) | 45 ± 4 (1.2) | 21.1 ± 2.9 (0.9) |
| 3704T | 1.6 ± 0.5 (2.4) | 17 ± 2 (3.2) | 41 ± 1 (1.4) | 19.1 ± 1.5 (1.0) |

[a]Site incidence immediately after 30-min exposure to 2 μM 4NQO. The amounts of damage detected after 1 or 4 μM 4NQO in cells from family members were relatively comparable to that seen after 2 μM (data not shown). [b]Mean ( ± s.e.) of 3 to 5 independent experiments. [c]Measured in cultures that were exposed to 2 μM 4NQO for 30 min and allowed to repair in the presence of $^3$H-dThd for 2 h. The number of grains above ⩾ 50 nuclei were scored for each slide (duplicate slides per strain). Results from two independent experiments are averaged for GM38, GM730, 3437T and 3701T fibroblasts. The five remaining strains were assayed in a single experiment. [d]Radioactivity in lysates of cultures incubated with $^3$H-4NQO (1 μM) for 20 min. [e]Quantified by the initial rate of NADH oxidation (expressed as nmol NADH oxidised/mg protein/min). [f]'*Normal Mean*' divided by value obtained for indicated strain.

**Figure 3** Accumulation of radiolabelled 4NQO in GM38 (●) and 3437T (○) fibroblasts. Data represent the mean ( ± range) of the values obtained for two experiments in which cell cultures were incubated with a range of drug concentrations for 10 and 20 min.

We reported previously (Mirzayans *et al.*, 1988*b*; Mirzayans & Paterson, 1991) that fibroblasts derived from subjects with the radiosensitive disorder ataxia-telangiectasia (A-T; complementation group A) are hypersensitive to killing by 4NQO and contain increased amounts of 4NQO-reductase activity. These same 4NQO-sensitive cells (e.g. strain AT3BI), however, were found to accumulate radiolabelled 4NQO at normal rates (Mirzayans *et al.*, 1988*b*). In contrast to that observed with A-T strains, we have demonstrated here that the 4NQO-resistant strains 3703T and 3704T perform 4NQO bioreduction normally but accumulate radiolabelled 4NQO to a reduced extent (Table III). These results imply that the rate of 4NQO retention in human fibroblasts is independent of cellular capacity to convert the parent compound to 4HAQO. Further studies are required to identify the various metabolic pathways and other factors that govern the accumulation of 4NQO in human cells, one or more of which may be aberrant in the 4NQO-resistant strains reported here.

In another earlier study (Marshall *et al.*, 1991*a*), strains 3437T, 3701T and 3702T were found to be more resistant to mitomycin C (MMC)-induced cell killing than two strains from unrelated normal donors. One of these MMC-resistant strains (3437T) showed normal colony-forming ability on exposure to the cross-linking agent *cis*-dichlorodiammine platinum II (Marshall *et al.*, 1989), implying that the enhanced resistance of these cells to MMC, which is also a cross-linking agent (Fujiwara, 1982), does not result from unusually rapid processing of DNA cross links. It should be noted that only those 4NQO-resistant strains harbouring reduced 4NQO-reductase activity (i.e. strains 3437T, 3701T and 3702T, but not 3703T or 3704T) also exhibited significant resistance to MMC. This is reminiscent of the results of Akamatsu and coworkers (1983) who reported that cells from certain patients with familial polyposis coli contained increased 4NQO-reductase activity and were hypersensitive to the lethal effects of both 4NQO and MMC. Together, these observations strongly implicate a common enzyme, presumably DT-diaphorase (Tsuda *et al.*, 1984), in the bioreduction of the two procarcinogens. In accord with this notion, the aerobic reduction of both 4NQO (Tsuda *et al.*, 1984) and MMC (Keyes *et al.*, 1989; Marshall *et al.*, 1991*b*) has been demonstrated to be inhibited by dicumarol, a potent (although not specific) inhibitor of DT-diaphorase (Ernster, 1967).

In our earlier work the level of DT-diaphorase activity was also determined for the five fibroblast strains in the polyposis/sarcoma family. Enzyme activities of 1820 and 6680 nmol mg protein/min were present in the two normal strains (GM38 and GM3529), whereas the activities in all five strains from the family members were found to be markedly lower than normal, ranging from 400–800 nmol mg protein/min in 3702T, 3703T and 3704T, to negligible levels (~30 nmol mg protein/min) in 3437T and 3701T (Marshall *et al.*, 1991*a*). In this communication cell sonicates of these same strains were shown to carry out 4NQO bioreduction at rates which are either comparable to (i.e. in 3703T, 3704T) or 40–60% lower than (i.e. in 3437T, 3701T, 3702T) the rates measured in cell preparations of four normal strains (Table III). These striking quantitative differences in the residual levels of DT-diaphorase *vs* 4NQO-reductase present in the same strains lend support to the hypothesis that a significant component of 4NQO-reductase activity residing in human cells is conferred by reductase(s) distinct from DT-diaphorase.

Dermal fibroblasts from patients with the cancer-prone diseases familial polyposis coli (Akamatsu *et al.*, 1983), A-T (Mirzayans *et al.*, 1988*b*; Mirzayans & Paterson, 1991) and dysplastic nevus syndrome (Mirzayans *et al.*, 1989*b*) typically display hypersensitivity to the cytotoxic action of 4NQO and this abnormal cellular response is accompanied by an enhanced capacity to bioreduce 4NQO to an activated derivative. Conversely, strains 3437T, 3701T and 3702T from three members of the cancer-prone family studied here exhibited decreased susceptibility to 4NQO (Table II) and contained reduced 4NQO-reductase activity (Table III). Factors that govern the expression of DT-diaphorase and other cellular reductases (Tsuda *et al.*, 1984) remain largely unknown. Their identification should not only establish a molecular basis for bioreduction of 4NQO and related compounds in human cells, but may also provide new insight into the nature of the fundamental genetic defects underlying various cancer-prone disorders. In addition, the discovery of other cancer-associated diseases characterised by anomalies in DT-diaphorase activity may lead to an improved understanding of the link between DT-diaphorase up-regulation [e.g., as seen in subjects with familial polyposis coli (Akamatsu *et al.*, 1983)] and down-regulation [e.g., as seen in the donors of 3437T and 3701T (Table III)] and the occurrence of specific cancers.

The observation that strain 3703T from a spousal control of the polyposis/sarcoma family displays enhanced resistance to 4NQO cytotoxicity (Table II) was unexpected. It should also be noted that strain 3704T from a disease-free family member in the cancer-prone lineage exhibits 4NQO-hyperresistance coupled with decreased 4NQO-reductase/DT-diaphorase activity (Tables II and III; Marshall *et al.*, 1991*a*). These findings, however, do not rule out the possibility that the propensity to develop cancer among family members and the impaired capacity of their cultured fibroblasts to bioreduce 4NQO are causally related, each a manifestation of the same primary genetic defect segregating in the family. In fact, this association between cellular reductase activity and tumorigenesis has been reported by other groups in diverse experimental systems (reviewed in Marshall *et al.*, 1991*a*). Moreover, an increasing body of evidence suggests that the principal defect in A-T may reside in a regulatory gene whose product may govern the expression of multiple homeostatic mechanisms including those controlling the bioreduction of 4NQO and other xenobiotics (Mirzayans *et al.*, 1989*a*). Identification of the primary genetic defect underlying cancer proneness in the family studied here may help to elucidate the functional significance of these homeostatic mechanisms.

Supported initially by Atomic Energy of Canada Limited and the US National Cancer Institute through Contract NO1-CP-21029 (Basic) with the Clinical and Environmental Epidemiology Branches, Bethesda, MD, and in the later stages by research grants from the Medical Research Council and National Cancer Institute of Canada and by generous donations from the Dr Herbert Meltzer Memorial Fund. M.C.P. is a Medical Scientist of the Alberta Heritage Foundation for Medical Research.

### References

AKAMATSU, N., MIYAKI, M., SUZUKI, K., ONO, T. & SASAKI, M.S. (1983). Mechanism of increased susceptibility to 4-nitroquinoline-1-oxide in cultured skin fibroblasts from patients with familial polyposis coli. *Mutat. Res.*, **120**, 173–180.

ARLETT, C.F. & HARCOURT, S.A. (1980). Survey of radiosensitivity in a variety of human cell strains. *Cancer Res.*, **40**, 926–932.

BROWN, A.J., FICKEL, T.H., CLEAVER, J.E., LOHMAN, P.H.M., WADE, M.H. & WATERS, R. (1979). Overlapping pathways for repair of damage from ultraviolet light and chemical carcinogens in human fibroblasts. *Cancer Res.*, **39**, 2522–2527.

CLEAVER, J.E. (1968). Defective repair replication of DNA in xeroderma pigmentosum. *Nature*, **218**, 652–656.

CLEAVER, J.E. (1989). Do we know the cause of xeroderma pigmentosum? *Carcinogenesis*, **11**, 875–882.

CLEAVER, J.E. & KRAEMER, K.H. (1989). Xeroderma pigmentosum. In *The Metabolic Basis of Inherited Disease*, Scriver, C.R., Beaudet, A.L., Sly, W.S. & Valle, D. (eds). 6th edn., Vol. II, pp. 2949–2971. McGraw-Hill: New York.

DESCHAVANNE, P.J., DEBIEU, D., FERTIL, B. & MALAISE, E.P. (1986). Re-evaluation of *in vitro* radiosensitivity of human fibroblasts of different genetic origins. *Int. J. Radiat. Biol.*, **50**, 279–293.

EDWARDS, S., FIELDING, S. & WATERS, R. (1987). The response to DNA damage induced by 4-nitroquinoline-1-oxide or its 3-methyl derivative in xeroderma pigmentosum fibroblasts belonging to different complementation groups: evidence for different epistasis groups involved in the repair of large adducts in human DNA. *Carcinogenesis*, **8**, 1071–1075.

ERNSTER, L. (1967). DT-diaphorase. *Methods Enzymol.*, **10**, 309–317.

ERNSTER, L. (1987). DT-diaphorase: a historical review. *Chem. Scrip.*, **27A**, 1–13.

FRAUMENI, J.F. Jr., VOGEL, C.L. & EASTON, J.M. (1968). Sarcomas and multiple polyposis in a kindred: a genetic variety of hereditary polyposis? *Arch Intern. Med.*, **121**, 57–61.

FUJIWARA, Y. (1982). Defective repair of mitomycin C crosslinks in Fanconi's anemia and loss in confluent normal human and xeroderma pigmentosum cells. *Biochim. Biophys. Acta*, **699**, 217–225.

GALIÈGUE-ZOUITINA, S., BAILLEUL, B. & LOUCHEUX-LEFEBVRE, M.-H. (1985). Adducts from *in vivo* action of the carcinogen 4-hydroxyaminoquinoline 1-oxide in rats and from *in vitro* reaction of 4-acetoxyaminoquinoline 1-oxide with DNA and polynucleotides. *Cancer Res.*, **45**, 520–525.

HANAWALT, P.C., COOPER, P.K., GANESAN, A.K. & SMITH, C.A. (1979). DNA repair in bacteria and mammalian cells. *Ann. Rev. Biochem.*, **48**, 783–836.

KEYES, S.R., ROCKWELL, S. & SARTORELLI, A.C. (1989). Modification of the metabolism and cytotoxicity of bioreductive alkylating agents by dicoumarol in aerobic and hypoxic murine tumour cells. *Cancer Res.*, **49**, 3310–3313.

LEHMANN, A.R., ARLETT, C.F., BROUGHTON, B.C., HARCOURT, S.A., STEINGRIMSDOTTIR, H., STEFANINI, M., TAYLOR, A.M.R., NATARAJAN, A.T., GREEN, S., KING, M.D., MACKIE, R.M., STEPHENSON, J.B.P. & TOLMIE, J.L. (1988). Trichothiodystrophy, a human DNA repair disorder with heterogeneity in the cellular response to ultraviolet light. *Cancer Res.*, **48**, 6090– 6096.

MAHER, V.M., DOMORADZKI, J., BHATTACHARYYA, N.P., TSUJIMURA, T., CORNER, R.C. & MCCORMICK, J.J. (1990). Alkylation damage, DNA repair and mutagenesis in human cells. *Mutat. Res.*, **233**, 235–245.

MARSHALL, R.S., PATERSON, M.C. & RAUTH, A.M. (1989). Deficient activation by a human cell strain leads to mitomycin resistance under aerobic but not hypoxic conditions. *Br. J. Cancer*, **59**, 341–346.

MARSHALL, R.S., PATERSON, M.C. & RAUTH, A.M. (1991*a*). DT-diaphorase activity and mitomycin C sensitivity in non-transformed cell strains derived from members of a cancer-prone family. *Carcinogenesis*, **12**, 1175–1180.

MARSHALL, R.S., PATERSON, M.C. & RAUTH, A.M. (1991*b*). Studies on the mechanism of resistance to mytomycin C and porfiromycin in a human cell strain derived from a cancer-prone individual. *Biochem. Pharmacol.*, **41**, 1351–1360.

MAYNE, L.V., MULLENDERS, L.H.F. & VAN ZEELAND, A.A. (1988). Cockayne's syndrome: a UV sensitive disorder with a defect in the repair of transcribing DNA but normal overall excision repair. In *Mechanisms and Consequences of DNA Damage Processing*, Friedberg, E. & Hanawalt, P. (eds). pp. 349–353. Alan R. Liss: New York.

MIRZAYANS, R. & WATERS, R. (1981). DNA damage and its repair in human normal or xeroderma pigmentosum fibroblasts treated with 4-nitroquinoline 1-oxide or its 3-methyl derivative. *Carcinogenesis*, **2**, 1359–1362.

MIRZAYANS, R., PATERSON, M.C. & WATERS, R. (1985). Defective repair of a class of 4NQO-induced alkali-labile DNA lesions in xeroderma pigmentosum complementation group A fibroblasts. *Carcinogenesis*, **6**, 555–559.

MIRZAYANS, R., WATERS, R. & PATERSON, M.C. (1988*a*). Induction and repair of DNA strand breaks and 1-$\beta$-D-arabinofuranosylcytosine-detectable sites in 40-75 kVp X-irradiated compared to $^{60}$Co $\gamma$-irradiated human cells lines. *Radiat. Res.*, **114**, 168–185.

MIRZAYANS, R., SABOUR, M. & PATERSON, M.C. (1988*b*). Enhanced bioreduction of 4-nitroquinoline 1-oxide by cultured ataxia telangiectasia fibroblasts. *Carcinogenesis*, **9**, 1711–1715.

MIRZAYANS, R., SMITH, B.P. & PATERSON, M.C. (1989*a*). Hypersensitivity to cell killing and faulty repair of 1-β-D-arabinofuranosylcytosine-detectable sites in human (ataxia-telangiectasia) fibroblasts treated with 4-nitroquinoline 1-oxide. *Cancer Res.*, **49**, 5523-5529.

MIRZAYANS, R., SABOUR, M. & PATERSON, M.C. (1989*b*). Bioreduction of 4-nitroquinoline 1-oxide in dysplastic nevus syndrome fibroblasts. *Matat. Res.*, **225**, 165-169.

MIRZAYANS, R. & PATERSON, M.C. (1991). Lack of correlation between hypersensitivity to cell killing and impaired inhibition of DNA synthesis in ataxia telangiectasia fibroblasts treated with 4-nitroquinoline 1-oxide. *Carcinogenesis*, **12**, 19-24.

MIRZAYANS, R., MIDDLESTADT, M.V. & PATERSON, M.C. (1992). Cytotoxic and mutagenic effects of methylnitrosourea in two human fetal fibroblast strains differing in $O^6$-methylguanine-DNA methyltransferase activity. *Carcinogenesis*, **13**, 1185-1190.

PATERSON, M.C. & SMITH, P.J. (1979). Ataxia telangiectasia: an inherited human disorder involving hypersensitivity to ionizing radiation and related DNA-damaging chemicals. *Ann. Rev. Genet.*, **13**, 291-318.

PATERSON, M.C., BECH-HANSEN, N.T., BLATTNER, W.A. & FRAUMENI, J.F. Jr. (1983). Survey of human hereditary and familial disorders for γ-ray response *in vitro*: occurrence of both cellular radiosensitivity and radioresistance in cancer-prone families. In *Radioprotectors and Anticarcinogens*, Nygaard, O.F. & Simic, M.G. (eds). pp. 615-638. Academic Press: New York.

PATERSON, M.C., GENTNER, N.E., MIDDLESTADT, M.V. & WEINFELD, M. (1984*a*). Cancer predisposition, carcinogen hypersensitivity, and aberrant DNA metabolism. *J. Cell. Physiol. Suppl.*, **3**, 45-62.

PATERSON, M.C., BECH-HANSEN, N.T., SMITH, P.J. & MULVIHILL, J.J. (1984*b*). Radiogenic neoplasia, cellular radiosensitivity, and faulty DNA repair. In *Radiation Carcinogenesis: Epidemiology and Biological Significance*, Boice, J.D. Jr. & Fraumeni, J.F. Jr. (eds), pp. 319-336. Raven Press: New York.

PATERSON, M.C., MIDDLESTADT, M.V., WEINFELD, M., MIRZAYANS, R. & GENTNER, N.E. (1986). Human cancer-prone disorders, abnormal carcinogen response and defective DNA metabolism. In *Radiation Carcinogenesis and DNA Alterations – NATO ASI Series A: Life Sciences*, Burns, F.J., Upton, A.C. & Silini, G. (eds). Vol. 124, pp. 471-498. Plenum Press: New York.

PATERSON, M.C., AUBIN, R.A., FOURNEY, R.M. & MIRZAYANS, R. (1989). Survey of post-γ ray colony-forming ability, DNA metabolism and oncogene status in nonmalignant fibroblast strains from cancer-prone families and individual cancer patients. In *14th L.H. Grey Conference on Low Dose Radiation: Biological Bases of Risk Assessment*, Baverstock, K.F. & Stather, J.W. (eds). pp. 227-239. Taylor & Francis: London.

RAMSAY, R.G., CHEN, P., IMRAY, F.P., KIDSON, C., LAVIN, M.F. & HOCKEY, A. (1982). Familial melanoma associated with dominant ultraviolet radiation sensitivity. *Cancer Res.*, **42**, 2909-2912.

REGAN, J.D. & SETLOW, R.B. (1974). Two forms of repair in the DNA of human cells damaged by chemical carcinogens and mutagens. *Cancer Res.*, **34**, 3318-3325.

SCHNEIDER, E.L., STANBRIDGE, E.J. & EPSTEIN, C.J. (1974). Incorporation of $^3$H-uridine and $^3$H-uracil into RNA: a simple technique for the detection of mycoplasma contamination of cultured cells. *Exp. Cell. Res.*, **84**, 311-318.

SMITH, P.J. & PATERSON, M.C. (1980). Defective DNA repair and increased lethality in ataxia telangiectasia cells exposed to 4-nitroquinoline-1-oxide. *Nature*, **287**, 747-749.

SUGIMURA, T., OKABE, K. & NAGAO, M. (1966). The metabolism of 4-nitroquinoline-1-oxide, a carcinogen. III. An enzyme catalyzing the conversion of 4-nitroquinoline-1-oxide to 4-hydroxyaminoquinoline-1-oxide in rat liver and hepatomas. *Cancer Res.*, **26**, 1717-1721.

TADA, M. & TADA, M. (1975). Seryl-tRNA synthetase and activation of the carcinogen 4-nitroquinoline-1-oxide. *Nature*, **255**, 510-512.

TARONE, R.E., SCUDIERO, D.A. & ROBBINS, J.H. (1983). Statistical methods for *in vitro* cell survival assays. *Mutat. Res.*, **111**, 79-96.

TSUDA, H., YOSHIDA, D. & MIZUSAKI, S. (1984). Caffeine inhibition of the metabolic activation of a carcinogen, 4-nitroquinoline-1-oxide, in cultured Chinese hamster cells. *Carcinogenesis*, **5**, 331-334.

WATERS, R. (1984). DNA repair tests in cultured mammalian cells. In *Mutagenicity Testing: A Practical Approach*, Venitt, S. & Parry, J.M. (eds). pp. 99-117. IRL Press: Oxford.

WATERS, R., MISHRA, N., BOUCK, N., DIMAYORCA, G. & REGAN, J.D. (1977). Partial inhibition of postreplication repair and enhanced frequency of chemical transformation in rat cells infected with leukemia virus. *Proc. Natl Acad. Sci. USA*, **74**, 238-242.

WEEKS, D.E., PATERSON, M.C., LANGE, K., ANDRAIS, B., DAVIS, R.C., YODER, F. & GATTI, R.A. (1991). Assessment of chronic γ radiosensitivity as an *in vitro* assay for heterozygote identification of ataxia-telangiectasia. *Radiat. Res.*, **128**, 90-99.

WEICHSELBAUM, R.R., NOVE, J. & LITTLE, J.B. (1980). X-ray sensitivity of fifty-three human diploid fibroblast cell strains from patients with characterized genetic disorders. *Cancer Res.*, **40**, 920-925.